# Obesity and Occupational Disparities in Urban China: Evidence from a Large-Scale Cross-Sectional Study

**DOI:** 10.3390/healthcare13172225

**Published:** 2025-09-05

**Authors:** Guoxi Zhang, Huyang Zhang, Gordon G. Liu, Leiyu Shi

**Affiliations:** 1 Department of Health Policy and Management, Johns Hopkins Bloomberg School of Public Health, Baltimore, MD 21205, USA; guoxi.zhang@jhu.edu; 2 National School of Development, Peking University, Beijing 100871, China; huyangzhang@pku.edu.cn; 3 Institute for Global Health and Development, Peking University, Beijing 100871, China

**Keywords:** overweight, obesity, body mass index, occupation, cross-sectional study, China

## Abstract

**Background/Objectives**: Obesity has become a global public health crisis, with China now ranking among the countries with the highest number of obese adults. Urban China faces a growing burden of obesity, but little is known about how obesity prevalence differs by occupation. This study aimed to assess the prevalence of overweight and obesity in 2022 among urban adults in northern and southern China, and to examine disparities across occupational groups. **Methods**: This study utilized cross-sectional data from 2022 health examinations to calculate the prevalence of overweight and obesity in different occupations in cities of southern and northern China. A logistic regression model was applied to analyze association between occupational categories and obesity, controlling for confounding factors like age, gender and region. **Results:** A total of 1,427,978 participants in 2022 were included. The aggregate prevalence of overweight and obesity was 30.78% and 7.90%, respectively. After being adjusted by logistic regression, the study showed that the highest adjusted odds ratio of overweight was observed in the Real Estate Industry sector with AOR 1.17 (1.15, 1.19), while the lowest odds ratio occurred in the Mining Industry sector, which was 0.90 (0.80, 1.01). The highest adjusted odds ratio of the prevalence of obesity was associated with the Production and Supply of Electricity, Heat, Gas, and Water sector (AOR: 1.92 (1.78, 2.07)), whereas the lowest odds ratio was observed in the Scientific Research and Technical Services sector. After categorizing occupations into four broad groups, the Blue-Collar group had the highest adjusted odds ratio of the prevalence of overweight (AOR: 1.07 (1.06, 1.08)), whereas the Sales/Office group exhibited the highest odds ratio of the prevalence of obesity (AOR: 1.37 (1.35, 1.39)). **Conclusions:** The prevalence of overweight and obesity differed substantially across occupational groups especially for obesity. More detailed and occupation-specific BMI management policies should be released to reduce obesity-related health inequalities in urban China.

## 1. Introduction

Obesity, defined by the World Health Organization as a body mass index (BMI) higher than 30 kg/m^2^ [1], is a risk factor for many noncommunicable diseases, including type 2 diabetes, hepatic steatosis and cardiovascular diseases, resulting in deaths worldwide [2,3,4,5]. Death related to BMI has increased markedly in recent years, reaching 3.71 million in 2021 from 1.46 million in 1990 [6]. Also, DALYs increased quickly from 1990 to 2021 especially among men [6,7]. In 2022, approximately 2.5 billion adults aged 18 years and older (43% of the global adult population, a substantial increase from 25% in 1990) were classified as overweight, including over 890 million with obesity [2]. Recent estimates from the NCD Risk Factor Collaboration indicated that by 2022, the number of obese adults globally had reached 504 million women and 374 million men [8]. China is among the top three countries in absolute numbers of obese individuals [8,9].

China has experienced a rapid rise in obesity in recent decades, making it a significant public health issue [10,11]. According to the national report on chronic diseases and nutrition, the combined prevalence of overweight and obesity in Chinese adults exceeded 50% by 2020 [12]. In China, 41% of adults are projected to have a high BMI (≥25 kg/m^2^) and 9% to be obese by 2025, with associated excess weight contributing to over 228,000 premature deaths and 8.5 million NCD-related DALYs annually [13]. This escalation poses a tremendous health and economic burden; one analysis projected that by 2030, approximately 65% of Chinese adults may be overweight or obese—up from around 25% in 2000 and 50% in 2020—affecting over 800 million people and incurring an estimated CNY 418 billion in annual healthcare costs [14].

Obesity arises from an energy imbalance driven by individual factors (diet, physical activity, genetics) operating within broader obesogenic environments [10]. Occupation serves as a critical social determinant of health, influencing energy expenditure, stress levels, work hours, and dietary access [15,16]. A study with 345 employees from the Information Technology sector found that prolonged sitting increased fat accumulation [17]. Adults spend a substantial portion of their lives at work, and previous research has shown that obesity prevalence can vary significantly across different industries and occupations [18,19,20]. In high-income countries, certain occupations with more sedentary work or job stress (e.g., office-based jobs, transportation) have been associated with higher obesity rates, whereas more physically demanding jobs can sometimes have lower rates, but most existing studies on occupational variations in obesity come from Western countries or use relatively small samples [19,21,22,23,24]. In China, there is a critical knowledge gap regarding how obesity is distributed across occupational groups and larger sample sizes [11,25,26,27,28,29]. Filling this gap is important for tailoring public health interventions, as different job sectors may face distinct challenges related to physical activity opportunities, diet, and work-related stress.

This study conducted a large-scale cross-sectional analysis of urban Chinese adults in 2022 to investigate the prevalence of overweight and obesity and their variation across occupational groups. Drawing on health examination data from Beijing, the capital of China and a major northern metropolis, and Zhejiang Province, an economically developed southern region, this study aimed to analyze the impact of different occupations on overweight and obesity, adjusting for relevant factors using logistic regression. The results are expected to provide an empirical foundation for designing occupation-specific strategies for obesity prevention and control, with implications for urban public health policy and workplace interventions in China.

## 2. Materials and Methods

This study was a cross-sectional analysis of anonymized health examination records collected in 2022 by a large private health screening provider in China. The dataset included adults who underwent routine health check-ups in two major regions: Beijing Municipality (a first-tier city in North China) and Zhejiang Province (an urbanized province in South China). These two regions were selected to reflect differing urban contexts in northern and southern China, respectively. All individuals aged ≥20 years who completed a health examination during the calendar year 2022 in the participating centers were eligible for inclusion. Entries with missing data on height, weight, sex, or occupation were excluded.

### 2.1. Definition and Measurement of Variables

#### 2.1.1. Overweight and Obesity

In this study, BMI was calculated from measured height (in meters) and weight (in kilograms) using the formula BMI = weight/(height)^2^ [2]. The dependent variables overweight and obesity were defined by BMI, using the World Health Organization (WHO) criteria [1], specifically:

(1) Overweight was defined as BMI ≥ 25 kg/m^2^ but <30 kg/m^2^;
(2) Obesity was defined as BMI ≥ 30 kg/m^2^.

In order to better understand the effect of different standards on the results, this study used both Chinese criteria to define overweight and obesity [30], specifically:

(1) Overweight was defined as BMI ≥ 24 kg/m^2^ but <28 kg/m^2^;
(2) Obesity was defined as BMI ≥ 28 kg/m^2^.

Results using Chinese criteria are detailed in the Supplementary Materials.

#### 2.1.2. Occupations and Categories

The occupational classifications for examinees in this study were based on the Industrial Classification for National Economic Activities (2017 Edition) issued by the National Bureau of Statistics of China [31]. The specific categories used were as follows: (1) Transportation, Storage, and Postal Services; (2) Accommodation and Catering Services; (3) Information Transmission, Software, and Information Technology Services; (4) Agriculture, Forestry, Animal Husbandry, and Fishery; (5) Manufacturing; (6) Health and Social Work; (7) Residential Services, Repair, and Other Services; (8) Construction; (9) Real Estate Industry; (10) Wholesale and Retail Trade; (11) Education; (12) Culture, Sports, and Entertainment; (13) Water Conservancy, Environment, and Public Facilities Management; (14) Production and Supply of Electricity, Heat, Gas, and Water; (15) Scientific Research and Technical Services; (16) Leasing and Business Services; (17) Mining Industry; (18) Financial Services; (19) Public Administration, Social Security, and Social Organizations; (20) International Organizations. The last two were excluded due to the absence of reliable occupational data.

To improve analytical power and facilitate interpretation, the 18 occupational categories were regrouped into four major classifications—Blue-Collar, Service, Sales/Office, and Management/Professional—based on occupational structures commonly used in prior studies examining the association between job characteristics and obesity risk [20,32,33,34,35]. The detailed mapping of original categories to these four groups is presented in Table 1.

#### 2.1.3. Covariates

To account for potential confounders, the analysis adjusted for a range of demographic and clinical covariates known to be associated with overweight and obesity. These included sex, age, and marital status, as well as two major metabolic risk factors: hypertension and hyperglycemia. Hypertension was defined according to the Chinese Guidelines for the Prevention and Treatment of Hypertension (2024 Revised Edition) as a systolic blood pressure (SBP) ≥ 140 mmHg and/or diastolic blood pressure (DBP) ≥ 90 mmHg [36]. Hyperglycemia was categorized following the Guideline for the prevention and treatment of diabetes mellitus in China (2024 edition): (1) prediabetes was defined as a fasting plasma glucose (FPG) level ≥ 6.1 mmol/L and <7.0 mmol/L, and (2) diabetes as an FPG level ≥ 7.0 mmol/L [37].

### 2.2. Statistical Analysis

During the basic descriptive statistical analysis of the data, for continuous variables, comparisons between groups were performed as follows: (1) Median comparisons (e.g., for variable age) used the non-parametric equality-of-medians test. (2) Mean comparisons used the *t*-test. For categorical variables (e.g., occupations, sex, region, marital status), comparisons of proportions between groups used the chi-square test. Subsequently, multivariable logistic regression models were employed to analyze the impact of different occupations on overweight and obesity. All data cleaning and statistical analyses were conducted using Stata 19 (StataCorp LLC, College Station, TX, USA) and all results are reproducible.

## 3. Results

### 3.1. Descriptive Characteristics of the Study Population

A total of 1,427,978 adults were included (Beijing n = 1,057,723; Zhejiang n = 370,255). Participants comprised 52.74% males and 47.26% females, with a median (interquartile range) age of 35 (29–44) years old. The overall prevalence of overweight and obesity were 30.78% and 7.90% using WHO criteria, while under Chinese criteria, the prevalences were 32.85% and 15.94%, respectively. Regarding occupational distribution, the Information Technology sector (26.52%) represented the largest proportion and Mining Industry sector (0.10%) the smallest. Among obesity-related complications, hypertension prevalence was 12.84%, prediabetes 5.38%, and diabetes 12.34%. Detailed data are summarized in Table 2.

### 3.2. Crude Prevalence of Overweight and Obesity by Region and Occupation

Table 3 showed the unadjusted prevalence of overweight by region and occupation. Overweight rates varied substantially across industries, from 39.50% in Production and Supply of Electricity, Heat, Gas, and Water sector to 28.27% in Information Transmission, Software, and Information Technology Services sector. Table 4 showed unadjusted prevalence of obesity by region and occupation, also varying widely (from 14.38% in Production and Supply of Electricity, Heat, Gas, and Water sector to 6.62% in Financial Services sector). Tables S1 and S2 illustrated the prevalence of overweight and obesity using Chinese criteria. These patterns highlight notable disparities in the prevalence of overweight and obesity across occupations.

### 3.3. Association Between Occupation and Overweight/Obesity

#### 3.3.1. Basic Result

As demonstrated in Table 3 and Table 4, significant variations in overweight and obesity prevalence exist across regions and occupational groups. Given the established associations with demographic factors including sex, age, and marital status, we employed multivariable logistic regression to analyze the occupation–overweight/obesity relationship while controlling for potential confounders.

Methodologically, we first ran unadjusted logistic regression models followed by fully adjusted models. The adjusted odds ratios (AOR) represented our benchmark findings, as reported in Table 5.

For overweight (columns 1–2), occupation demonstrated significant effects. Using the Information Transmission, Software, and Information Technology Services sector, which had the largest sample size as a reference, most occupations showed elevated overweight odds ratios. The highest AOR occurred in the Real Estate sector (AOR: 1.17 (1.15, 1.19)), while the Mining Industry sector exhibited the lowest (AOR:0.90 (0.80, 1.01)). Regarding obesity (columns 3–4), the Production and Supply of Electricity, Heat, Gas, and Water sector demonstrated the highest adjusted odds ratio (AOR: 1.92 (1.78, 2.07)), with the Scientific Research and Technical Services sector showing the lowest (AOR:1.04 (0.84, 1.28)).

After examining the distribution of our main variable, occupation, clear disparities in the prevalence of overweight and obesity were observed. The prevalence was lower in the southern region compared to the northern region. For instance, the adjusted odds ratio (AOR) for overweight in the south was 0.83 (0.82, 0.83). Men showed a significantly higher prevalence of overweight than women, with an AOR of 2.76 (2.74, 2.78). Different age groups also exhibited distinct patterns in overweight and obesity. For overweight, the 50–59 age group had the highest risk, with an AOR of 2.48 (2.44, 2.51) compared to the 20–29 age group. In contrast, for obesity, the 30–39 age group showed the highest risk, with an AOR of 1.06 (1.04–1.08) relative to the 20–29 age group.

Regression analyses using Chinese criteria for defining overweight and obesity yielded generally consistent overall results, though some subtle differences were noted. Most notably, the prevalence of obesity varied considerably across occupational groups, with the highest AOR being 1.74 (1.64, 1.84). Consistent with above findings, southern region had lower rates than northern region, and men had higher rates than women. Regarding age-specific risks, the 60–69 group had the highest risk for overweight, while the 40–49 group had the highest risk for obesity. For detailed results, please refer to Table S3 in the Supplemental Materials.

**Figure 1:**
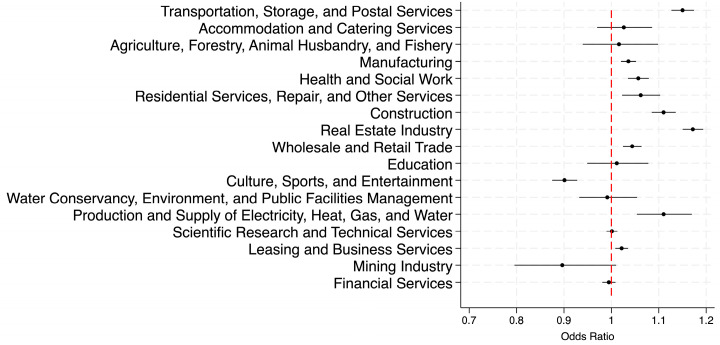
Adjusted odds ratio of overweight across occupations.

**Figure 2:**
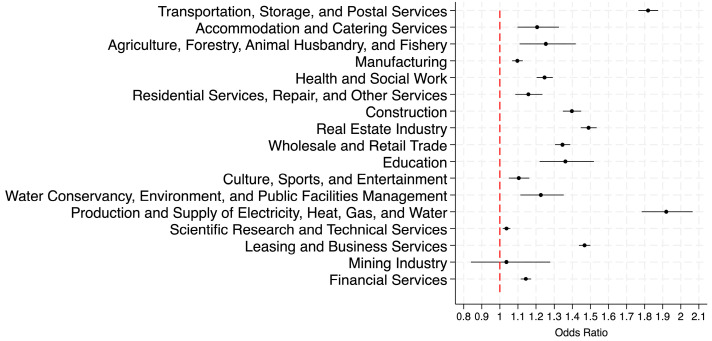
Adjusted odds ratio of obesity across occupations.

To better visualize the occupational variations in odds ratios presented in Table 5, Figure 1 and Figure 2 graphically depict these results, revealing substantial disparities in overweight and obesity prevalence across occupations. A comparison of the two figures below revealed that occupational disparities in obesity prevalence were substantially more pronounced than those in overweight prevalence. These findings underscore the importance of developing and implementing occupation-specific strategies for effective obesity management. Figures S1 and S2 present almost the same patterns under Chinese criteria.

#### 3.3.2. Results of Occupational Subclassification

As detailed in the Methods section, it was decided that the number of occupational categories (18 in total) was excessive and they were further consolidated into four broader groups to allow us to better understand the kinds of occupations: Blue-Collar, Sales/Office, Service, and Management/Professional. This study now used logistic regression to analyze the difference between these four groups; the Management/Professional sector with the largest sample size served as the reference group. Key results are presented in Table 6.

Columns 1 and 2 showed that among these four occupational groups, the Blue-Collar sector had the highest adjusted odds ratio of the prevalence of overweight. However, the regression results for obesity (columns 3 and 4) revealed that the Sales/Office group exhibited the highest adjusted odds ratio of the prevalence of obesity. Table S4 showed that the Management/Professional sector had the highest adjusted odds ratio of the prevalence of overweight and the Sales/Office sector had the highest adjusted odds ratio of the prevalence of obesity, respectively.

#### 3.3.3. Results of Region and Age Subgroups

**Figure 3:**
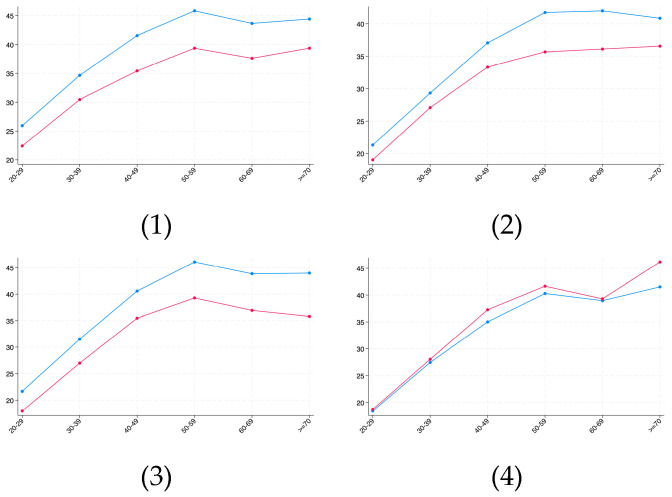
Prevalence of overweight across region and age. The blue line denotes north and the red line denotes south. (**1**) Overweight prevalence in Blue-collar sector. (**2**) Overweight prevalence in Management/Professional sector. (**3**) Overweight prevalence in Sales/Office sector. (**4**) Overweight prevalence in Service sector.

**Figure 4:**
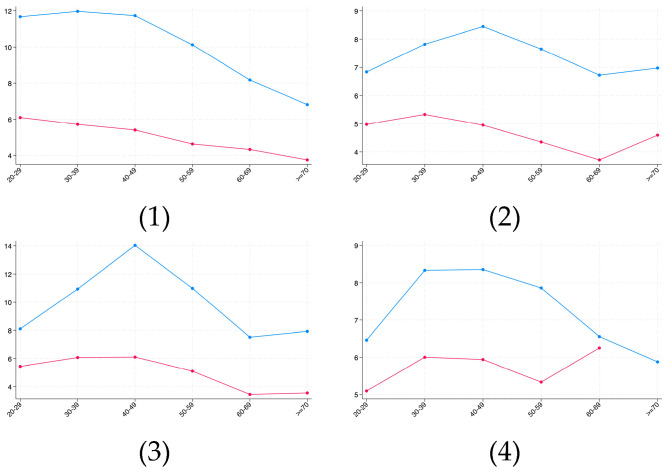
Prevalence of obesity across region and age. The blue line denotes north and the red line denotes south. (**1**) Overweight prevalence in Blue-collar sector. (**2**) Overweight prevalence in Management/Professional sector. (**3**) Overweight prevalence in Sales/Office sector. (**4**) Overweight prevalence in Service sector.

As shown in Table 5, this study observed that age and region significantly influenced the prevalence of overweight and obesity. Therefore, to address this, this study conducted further subgroup analyses stratified by regions and age groups. The differences in overweight and obesity prevalence across regions and age groups are presented in Figure 3 and Figure 4, respectively.

Figure 3 (Overweight Prevalence): The trends across the four panels (subgroups) were largely consistent, where overweight prevalence was higher in northern regions than in southern regions, and increased with age across all subgroups, with the Service sector showing minimal difference.

Figure 4 (Obesity Prevalence): The trends for obesity prevalence revealed markedly different patterns compared to Figure 3. Notably, within the Blue-Collar group, obesity prevalence decreased with increasing age. This divergence may be attributable to occupation-specific factors.

Figures S3 and S4 show minimal differences compared to Figure 3 and Figure 4. For instance, the prevalence of obesity decreased with increasing age in the Blue-Collar sector in the south region under WHO criteria but increased before the age of 50 years old in the south region under Chinese criteria.

## 4. Discussion

In this large-scale cross-sectional study of 1,427,978 urban Chinese adults (2022), sample demographics closely mirrored national population structures reported in prior literature, supporting the representativeness of our findings [25]. We observed an overall overweight prevalence of 30.78% and obesity prevalence of 7.90% using WHO criteria, and these rates align with China’s documented rising obesity trends [8,9,10,13]. Consistent with previous studies among Chinese workers, subgroup analyses revealed higher overweight/obesity prevalence among males versus females and in northern versus southern regions [25,27]. Men exhibited consistently higher odds ratio of being overweight/obesity across all occupational categories and both geographic regions, even after adjusting for age, marital status, hypertension, and hyperglycemia, a pattern that reinforces well-established evidence from national and international research attributing gender disparities in adiposity to sex-specific physiological factors such as visceral fat accumulation and hormonal regulation, as well as to sociocultural determinants including differential norms around physical activity, dietary restraint, and health-related help-seeking behaviors [26,27,38,39,40,41]. The significantly higher obesity prevalence observed in Beijing compared to Zhejiang likely reflects regional contextual influences—including dietary habits, occupational structures, and urban infrastructure—and is consistent with prior national evidence indicating that northern Chinese populations generally exhibit higher BMI and cardiometabolic risk due to greater energy intake, higher animal fat consumption, and environmental constraints on physical activity [25,27,38].

Regarding occupational disparities in overweight and obesity, this study found that while the Blue-Collar group exhibited the highest prevalence of overweight; its adjusted odds ratio (AOR = 1.07, 95% CI: 1.06–1.08) showed only marginal elevation compared to other occupational categories. Conversely, the Sales/Office group demonstrated the highest obesity prevalence with a clinically significant AOR of 1.37 (95% CI: 1.35–1.39). Occupational disparities in obesity likely arise from a complex interplay of factors, including physical activity demands, work-related stress, and dietary environments, whereby Blue-Collar workers—despite engaging in physically demanding labor—may experience elevated caloric intake due to fatigue-induced overeating, irregular schedules, and reliance on energy-dense convenience foods, while White-Collar employees are more prone to sedentary behavior, chronic psychological stress, and extended working hours that limit physical activity and promote stress-related eating, all of which are further compounded by occupation-associated socioeconomic gradients such as differences in income, education, and health literacy [15,34,42].

However, substantial cross-national variations exist in overweight/obesity prevalence. A 2011 US occupational survey reported an obesity prevalence of 27.6% among American workers—significantly higher than 7.90% in this study [19]. Similarly, UK statistics for health sector workers showed a 24.18% obesity rate, also markedly exceeding 7.53% prevalence in the Health and Social Work sector [43]. These findings collectively highlight China’s distinct obesity profile compared to Western nations, particularly regarding occupation-specific patterns. This divergence underscores the critical need to establish China-specific epidemiological databases for overweight/obesity with locally relevant occupational classifications. Such data infrastructure will enable advanced meaningful international comparisons.

From the policy management prospective, China has introduced relevant policies advocating for weight management guidance targeting specific groups such as occupational populations [44]. Meanwhile, there is a lack of empirically proven and effective methods. For instance, a systematic review had indicated that workplace interventions did not lead to a reduction in prolonged sitting [45]. These suggest the need to accelerate the integration of weight management into various policies and underscore the importance of generating more empirical evidence on effective workplace weight-loss interventions to inform future policy development in China.

The primary limitation of this study lies in its reliance on the China Standard Industrial Classification for occupational categorization. While this framework provides standardized groupings, it lacks granularity to capture further nuance within industries, and data on these internal variations were not accessible for this analysis. Furthermore, this study focused on BMI as the principal measure, which does not capture abdominal obesity—a central feature of metabolic syndrome and a key risk factor for cardiovascular disease and type 2 diabetes. The absence of waist circumference measurements limits the assessment of adiposity distribution and its metabolic implications. In addition, although hypertension and diabetes were considered, other components of metabolic syndrome—such as lipid disorders—were not incorporated into the analysis. This omission may affect the comprehensiveness of cardiometabolic risk profiling. Another limitation is the geographic imbalance in the study population, with a disproportionate representation of residents from Beijing compared to other cities across southern and northern China, which may affect the generalizability of the findings.

Nevertheless, this study possesses significant strengths including a large sample size, objectively measured height and weight data from health examinations, and the application of a standardized industry classification system aligned with the National Bureau of Statistics—features that facilitate policy alignment. The prevalence rates of overweight and obesity across these 18 occupational categories, as reported here, provide a valuable reference for future comparative research. This work aims to contribute to the ongoing scholarly discourse on obesity management within China’s workforce and to inform evidence-based recommendations for relevant public health policies.

## 5. Conclusions

This study, using large-scale health examination data, revealed significant disparities in the prevalence of overweight and obesity across occupational groups in urban China. The findings underscore the need to incorporate occupation-specific considerations into obesity prevention efforts and public health policies and also highlight a gap in the literature of evidence-based effective working environmental interventions.

## Figures and Tables

**Table 1:** Occupations and categories.

Number	Occupation in English	Categories
1	Transportation, Storage, and Postal Services	Blue-Collar
2	Accommodation and Catering Services	Service
3	Information Transmission, Software, and Information Technology Services	Management/Professional
4	Agriculture, Forestry, Animal Husbandry, and Fishery	Blue-Collar
5	Manufacturing	Blue-Collar
6	Health and Social Work	Management/Professional
7	Residential Services, Repair, and Other Services	Service
8	Construction	Blue-Collar
9	Real Estate Industry	Sales/Office
10	Wholesale and Retail Trade	Sales/Office
11	Education	Management/Professional
12	Culture, Sports, and Entertainment	Service
13	Water Conservancy, Environment, and Public Facilities Management	Blue-Collar
14	Production and Supply of Electricity, Heat, Gas, and Water	Blue-Collar
15	Scientific Research and Technical Services	Management/Professional
16	Leasing and Business Services	Sales/Office
17	Mining Industry	Blue-Collar
18	Financial Services	Management/Professional

**Table 2:** Participant demographics and characteristics.

Characteristics	Overall n = 1,427,978	North n = 1,057,723	South n = 370,255	*p*-Value
Age (year), median (IQR)	35 (29–44)	35 (29–45)	32 (27–41)	<0.001
Male, n (%)	753,102 (52.74)	557,144 (52.67)	195,958 (52.93)	0.0084
Married, n (%)	496,339 (34.76)	352,736 (33.35)	143,603 (38.78)	<0.001
Occupations, n (%)				<0.001
Transportation, Storage, and Postal Services	49,965 (3.50)	42,438 (4.01)	7527 (2.03)	
Accommodation and Catering Services	5986 (0.42)	5338 (0.50)	648 (0.18)	
Information Transmission, Software, and Information Technology Services	378,698 (26.52)	255,224 (24.13)	123,474 (33.35)	
Agriculture, Forestry, Animal Husbandry, and Fishery	3017 (0.21)	2850 (0.27)	167 (0.05)	
Manufacturing	110,731 (7.75)	68,048 (6.43)	42,683 (11.53)	
Health and Social Work	53,014 (3.71)	32,761 (3.10)	20,253 (5.47)	
Residential Services, Repair, and Other Services	13,990 (0.98)	11,049 (1.04)	2941 (0.79)	
Construction	39,354 (2.76)	32,053 (3.03)	7301 (1.97)	
Real Estate Industry	65,961 (4.62)	47,487 (4.49)	18,474 (4.99)	
Wholesale and Retail Trade	68,349 (4.79)	42,775 (4.04)	25,574 (6.91)	
Education	4931 (0.35)	4595 (0.43)	336 (0.09)	
Culture, Sports, and Entertainment	26,323 (1.84)	23,828 (2.25)	2495 (0.67)	
Water Conservancy, Environment, and Public Facilities Management	4830 (0.34)	4590 (0.43)	240 (0.06)	
Production and Supply of Electricity, Heat, Gas, and Water	6650 (0.47)	6354 (0.60)	296 (0.08)	
Scientific Research and Technical Services	274,217 (19.20)	222,593 (21.04)	51,624 (13.94)	
Leasing and Business Services	172,556 (12.08)	139,799 (13.22)	32,757 (8.85)	
Mining Industry	1378 (0.10)	1371 (0.13)	7 (0.00)	
Financial Services	148,028 (10.37)	114,570 (10.83)	33,458 (9.04)	
Overweight/Obesity				
Overweight (WHO criteria), %	30.78	32.22	26.67	<0.001
Obesity (WHO criteria), %	7.90	8.83	5.24	<0.001
Overweight (Chinese criteria), %	32.85	33.67	30.49	<0.001
Obesity (Chinese criteria), %	15.94	17.49	11.52	<0.001
Complications				
High blood pressure, %	12.84	13.48	11.01	<0.001
Diabetes, %	12.34	12.30	12.44	0.0259
Prediabetes, %	5.38	5.48	5.08	<0.001

**Table 3:** Prevalence of overweight by region and occupation.

Occupational Category	Overall n = 1,427,978	North n = 1,057,723	South n = 370,255	*p*-Value
Transportation, Storage, and Postal Services	36.89 (48.25)	38.34 (48.62)	28.74 (45.26)	<0.001
Accommodation and Catering Services	35.13 (47.74)	35.65 (47.90)	30.86 (46.23)	0.0160
Information Transmission, Software, and Information Technology Services	28.27 (45.03)	29.44 (45.58)	25.86 (43.79)	<0.001
Agriculture, Forestry, Animal Husbandry, and Fishery	38.05 (48.56)	38.63 (48.70)	28.14 (45.11)	0.0067
Manufacturing	31.79 (46.57)	34.02 (47.38)	28.23 (45.01)	<0.001
Health and Social Work	31.09 (46.29)	33.03 (47.03)	27.97 (44.88)	<0.001
Residential Services, Repair, and Other Services	32.47 (46.83)	32.15 (46.71)	33.70 (47.28)	0.1111
Construction	37.43 (48.40)	38.60 (48.68)	32.28 (46.76)	<0.001
Real Estate Industry	36.43 (48.12)	38.34 (48.62)	31.52 (46.46)	<0.001
Wholesale and Retail Trade	30.73 (46.14)	34.72 (47.61)	24.07 (42.75)	<0.001
Education	31.03 (46.27)	31.43 (46.43)	25.60 (43.70)	0.0257
Culture, Sports, and Entertainment	28.43 (45.11)	29.08 (45.42)	22.16 (41.54)	<0.001
Water Conservancy, Environment, and Public Facilities Management	38.05 (48.56)	38.26 (48.61)	34.17 (47.53)	0.2033
Production and Supply of Electricity, Heat, Gas, and Water	39.50 (48.89)	40.02 (49.00)	28.38 (45.16)	<0.001
Scientific Research and Technical Services	29.52 (45.61)	30.41 (46.00)	25.70 (43.7)	<0.001
Leasing and Business Services	32.12 (46.69)	33.46 (47.18)	26.40 (44.08)	<0.001
Mining Industry	31.06 (46.29)	31.15 (46.33)	14.29 (37.80)	0.3367
Financial Services	30.05 (45.85)	31.29 (46.37)	25.79 (43.75)	<0.001

Notes: Number of cases and corresponding prevalence rate (%).

**Table 4:** Prevalence of obesity by region and occupation.

Occupational Category	Overall n = 1,427,978	North n = 1,057,723	South n = 370,255	*p*-Value
Transportation, Storage, and Postal Services	13.84 (34.53)	15.05 (35.75)	7.00 (25.52)	<0.001
Accommodation and Catering Services	8.75 (28.26)	8.86 (28.42)	7.87 (26.95)	0.3995
Information Transmission, Software, and Information Technology Services	6.71 (25.02)	7.58 (26.47)	4.93 (21.64)	<0.001
Agriculture, Forestry, Animal Husbandry, and Fishery	10.28 (30.37)	10.21 (30.28)	11.38 (31.85)	0.6295
Manufacturing	7.43 (26.22)	8.90 (28.48)	5.07 (21.95)	<0.001
Health and Social Work	7.53 (26.39)	8.80 (28.33)	5.47 (22.74)	<0.001
Residential Services, Repair, and Other Services	7.93 (27.02)	8.52 (27.91)	5.71 (23.21)	<0.001
Construction	10.61 (30.80)	11.22 (31.56)	7.92 (27.00)	<0.001
Real Estate Industry	10.52 (30.68)	11.60 (32.02)	7.74 (26.72)	<0.001
Wholesale and Retail Trade	8.25 (27.51)	10.18 (30.23)	5.02 (21.85)	<0.001
Education	7.63 (26.54)	7.94 (27.04)	3.27 (17.82)	0.0018
Culture, Sports, and Entertainment	6.80 (25.18)	7.02 (25.54)	4.73 (21.23)	<0.001
Water Conservancy, Environment, and Public Facilities Management	10.39 (30.52)	10.57 (30.74)	7.08 (25.71)	0.0848
Production and Supply of Electricity, Heat, Gas, and Water	14.38 (35.09)	14.73 (35.44)	6.76 (25.14)	<0.001
Scientific Research and Technical Services	6.92 (25.38)	7.31 (26.04)	5.23 (22.27)	<0.001
Leasing and Business Services	9.85 (29.80)	10.98 (31.26)	5.01 (21.82)	<0.001
Mining Industry	7.18 (25.83)	7.22 (25.89)	NA	NA
Financial Services	6.62 (24.87)	7.28 (25.98)	4.37 (20.44)	<0.001

Notes: Number of cases and corresponding prevalence rate (%). NA indicates missing data due to insufficient sample size.

**Table 5:** Association between occupation and overweight/obesity.

Occupational Category	Overweight		Obesity	
	OR (95% CI)	AOR (95% CI)	OR (95% CI)	AOR (95% CI)
Occupations				
Transportation, Storage, and Postal Services	1.48 *** (1.45, 1.51)	1.15 *** (1.13, 1.17)	2.23 *** (2.17, 2.30)	1.82 *** (1.76, 1.87)
Accommodation and Catering Services	1.37 *** (1.30, 1.45)	1.03 (0.97, 1.09)	1.33 *** (1.22, 1.46)	1.21 *** (1.10, 1.33)
Agriculture, Forestry, Animal Husbandry, and Fishery	1.56 *** (1.45, 1.68)	1.02 (0.94, 1.1)	1.59 *** (1.41, 1.79)	1.25 *** (1.11, 1.42)
Manufacturing	1.18 *** (1.17, 1.20)	1.04 *** (1.02, 1.05)	1.11 *** (1.09, 1.14)	1.10 *** (1.07, 1.13)
Health and Social Work	1.14 *** (1.12, 1.17)	1.06 *** (1.04, 1.08)	1.13 *** (1.09, 1.17)	1.25 *** (1.20, 1.29)
Residential Services, Repair, and Other Services	1.22 *** (1.18, 1.26)	1.06 *** (1.02, 1.1)	1.20 *** (1.12, 1.27)	1.16 *** (1.09, 1.24)
Construction	1.52 *** (1.49, 1.55)	1.11 *** (1.09, 1.14)	1.65 *** (1.59, 1.71)	1.40 *** (1.35, 1.45)
Real Estate Industry	1.45 *** (1.43, 1.48)	1.17 *** (1.15, 1.19)	1.63 *** (1.59, 1.68)	1.49 *** (1.45, 1.53)
Wholesale and Retail Trade	1.13 *** (1.11, 1.15)	1.04 *** (1.02, 1.06)	1.25 *** (1.21, 1.29)	1.35 *** (1.3, 1.39)
Education	1.14 *** (1.07, 1.21)	1.01 (0.95, 1.08)	1.15 ** (1.03, 1.28)	1.36 *** (1.22, 1.52)
Culture, Sports, and Entertainment	1.01 (0.98, 1.04)	0.90 *** (0.87, 0.93)	1.01 (0.96, 1.07)	1.10 *** (1.05, 1.16)
Water Conservancy, Environment, and Public Facilities Management	1.56 *** (1.47, 1.65)	0.99 (0.93, 1.05)	1.61 *** (1.47, 1.77)	1.23 *** (1.11, 1.35)
Production and Supply of Electricity, Heat, Gas, and Water	1.66 *** (1.58, 1.74)	1.11 *** (1.05, 1.17)	2.33 *** (2.18, 2.50)	1.92 *** (1.78, 2.07)
Scientific Research and Technical Services	1.06 *** (1.05, 1.07)	1.00 (0.99, 1.01)	1.03 *** (1.01, 1.05)	1.04 *** (1.02, 1.06)
Leasing and Business Services	1.20 *** (1.19, 1.22)	1.02 *** (1.01, 1.04)	1.52 *** (1.49, 1.55)	1.47 *** (1.44, 1.50)
Mining Industry	1.14 ** (1.02, 1.28)	0.90 * (0.80, 1.01)	1.08 (0.88, 1.32)	1.04 (0.84, 1.28)
Financial Services	1.09 *** (1.08, 1.10)	0.99 (0.98, 1.01)	0.99 (0.96, 1.01)	1.14 *** (1.12, 1.17)
Male	NO	2.76 *** (2.74, 2.78)	NO	2.11 *** (2.08, 2.14)
Married	NO	1.06 *** (1.05, 1.07)	NO	0.99 (0.98, 1.00)
Age group	NO		NO	
30–39		1.50 *** (1.48, 1.51)		1.06 *** (1.04, 1.08)
40–49		2.11 *** (2.08, 2.13)		0.95 *** (0.93, 0.97)
50–59		2.48 *** (2.44, 2.51)		0.60 *** (0.59, 0.62)
60–69		2.42 *** (2.38, 2.46)		0.43 *** (0.42, 0.45)
>=70		2.15 *** (2.08, 2.21)		0.33 *** (0.31, 0.35)
South	NO	0.83 *** (0.82, 0.83)	NO	0.58 *** (0.57, 0.59)
Associated complications	NO		NO	
High blood pressure		1.44 *** (1.43, 1.46)		3.64 *** (3.58, 3.69)
Prediabetes		1.43 *** (1.41, 1.45)		2.51 *** (2.45, 2.56)
Diabetes		1.17 *** (1.16, 1.18)		1.95 *** (1.91, 1.98)
Constant	0.39 *** (0.39, 0.40)	0.14 *** (0.14, 0.15)	0.07 *** (0.07, 0.07)	0.04 *** (0.04, 0.04)
R^2^	0.0027	0.0719	0.0073	0.0896
Observations	1,427,978	1,427,978	1,427,978	1,427,978

Notes: Regressions of column 2 and 4 controlled for gender, marital status, age, region and associated complications. *** *p* < 0.01, ** *p* < 0.05, * *p* < 0.1.

**Table 6:** Association between occupation and obesity or overweight.

Occupational Category	Overweight		Obesity	
	OR (95% CI)	OR (95% CI)	OR (95% CI)	OR (95% CI)
Blue-Collar	1.28 *** (1.26, 1.29)	1.07 *** (1.06, 1.08)	1.49 *** (1.46, 1.51)	1.29 *** (1.27, 1.31)
Sales/Office	1.18 *** (1.17, 1.19)	1.05 *** (1.05, 1.06)	1.46 *** (1.44, 1.48)	1.37 *** (1.35, 1.39)
Service	1.07 *** (1.05, 1.09)	0.96 *** (0.94, 0.98)	1.09 *** (1.05, 1.13)	1.07 *** (1.04, 1.12)
Other covariates	NO	YES	NO	YES
R^2^	0.0017	0.0717	0.0046	0.0882
Observations	1,427,978	1,427,978	1,427,978	1,427,978

Management/Professional as the control group. *** *p* < 0.01.

## Data Availability

Restrictions apply to the availability of these data. Data were obtained from the iKang Healthcare Group and are available with the permission of the iKang Healthcare Group.

## Supplementary Materials

The following supporting information can be downloaded at https://www.mdpi.com/article/10.3390/healthcare13172225/s1: Table S1. Prevalence of Overweight by Region and Occupation (Chinese Criteria); Table S2. Prevalence of Obesity by Region and Occupation (Chinese Criteria); Table S3. Occupational Patterns in Overweight and Obesity (Chinese Criteria); Table S4. Association between Occupation and Obesity or Overweight (Chinese Criteria); Figure S1. Adjusted Odds Ratio of Overweight across Occupations (Chinese Criteria); Figure S2. Adjusted Odds Ratio of Obesity across Occupations (Chinese Criteria); Figure S3. Prevalence of Overweight across Region and Age (Chinese Criteria); Figure S4. Prevalence of Obesity across Region and Age (Chinese Criteria).
