# Correction: Zhang et al. Obesity and Occupational Disparities in Urban China: Evidence from a Large-Scale Cross-Sectional Study. *Healthcare* 2025, *13*, 2225

**DOI:** 10.3390/healthcare14050671

**Published:** 2026-03-06

**Authors:** Guoxi Zhang, Huyang Zhang, Gordon G. Liu, Leiyu Shi

**Affiliations:** 1 Department of Health Policy and Management, Johns Hopkins Bloomberg School of Public Health, Baltimore, MD 21205, USA; guoxi.zhang@jhu.edu; 2 National School of Development, Peking University, Beijing 100871, China; huyangzhang@pku.edu.cn; 3 Institute for Global Health and Development, Peking University, Beijing 100871, China

**Error in Figure/Table**

In the original publication [1], there was a mistake in the legend for Figure 4. Prevalence of obesity across region and age. All instances of “Overweight” should be corrected to “Obesity”. Below is the correct legend:

**Figure 4.** Prevalence of obesity across region and age. The blue line denotes north and the red line denotes south. (**1**) Obesity prevalence in Blue-collar sector. (**2**) Obesity prevalence in Management/Professional sector. (**3**) Obesity prevalence in Sales/Office sector. (**4**) Obesity prevalence in Service sector.

The footnote for Table 3 “Number of cases and corresponding prevalence rate (%)”, should be corrected to “Prevalence rate (%) and corresponding standard deviation”. 

The footnote for Table 4, “Number of cases and corresponding prevalence rate (%). NA indicates missing data due to insufficient sample size.”, should be corrected to “Prevalence rate (%) and corresponding standard deviation. NA indicates missing data due to insufficient sample size.” 

There was a mistake in Table 6. Association between occupation and obesity or overweight. In the heading in Column 3 and Column 4, “OR” should be corrected to “AOR”. The corrected version of Table 6 appears below. 

**Text Correction**

In Section 3.3.1, Paragraph 3, a confidence interval should be adjusted to remain consistent with Table 5. “AOR: 1.04 (0.84, 1.28)” should be corrected to “AOR 1.04 (1.02, 1.06)”. The full paragraph should read as below:

For overweight (columns 1–2), occupation demonstrated significant effects. Using the Information Transmission, Software, and Information Technology Services sector, which had the largest sample size as a reference, most occupations showed elevated overweight odds ratios. The highest AOR occurred in the Real Estate sector (AOR: 1.17 (1.15, 1.19)), while the Mining Industry sector exhibited the lowest (AOR:0.90 (0.80, 1.01)). Regarding obesity (columns 3–4), the Production and Supply of Electricity, Heat, Gas, and Water sector demonstrated the highest adjusted odds ratio (AOR: 1.92 (1.78, 2.07)), with the Scientific Research and Technical Services sector showing the lowest (AOR 1.04 (1.02, 1.06)).

The authors state that the scientific conclusions are unaffected. All corrections above were approved by the Academic Editor. The original publication has also been updated.

## Figures and Tables

**Table 6:** Association between occupation and obesity or overweight.

Occupational Category	Overweight		Obesity	
	OR (95% CI)	AOR (95% CI)	OR (95% CI)	AOR (95% CI)
Blue-Collar	1.28 *** (1.26, 1.29)	1.07 *** (1.06, 1.08)	1.49 *** (1.46, 1.51)	1.29 *** (1.27, 1.31)
Sales/Office	1.18 *** (1.17, 1.19)	1.05 *** (1.05, 1.06)	1.46 *** (1.44, 1.48)	1.37 *** (1.35, 1.39)
Service	1.07 *** (1.05, 1.09)	0.96 *** (0.94, 0.98)	1.09 *** (1.05, 1.13)	1.07 *** (1.04, 1.12)
Other covariates	NO	YES	NO	YES
R^2^	0.0017	0.0717	0.0046	0.0882
Observations	1,427,978	1,427,978	1,427,978	1,427,978

Management/Professional as the control group. *** *p* < 0.01.
