# Supplementary material for: Obesity and Occupational Disparities in Urban China: Evidence from a Large-Scale Cross-Sectional Study
Source: Healthcare (Basel). 2025 Sep 5;13(17):2225. doi: 10.3390/healthcare13172225 (PMC12428261; doi:10.3390/healthcare13172225)
Supplement: Supplementary file 1 [file healthcare-13-02225-s001.zip › healthcare-3757289-supplementary.pdf]

Supplementary Online Content

Table S1. Prevalence of Overweight by Region and Occupation (Chinese Criteria).

Table S2. Prevalence of Obesity by Region and Occupation (Chinese Criteria).

Table S3. Occupational Patterns in Overweight and Obesity (Chinese Criteria).

Table S4. Association between Occupation and Obesity or Overweight (Chinese Criteria).

Figure S1. Adjusted Odds Ratio of Overweight across Occupations (Chinese Criteria).

Figure S2. Adjusted Odds Ratio of Obesity across Occupations (Chinese Criteria).

Figure S3. Prevalence of Overweight across Region and Age (Chinese Criteria).

Figure S4. Prevalence of Obesity across Region and Age (Chinese Criteria).

This supplementary material was provided by the authors to give readers the results with overweight and obesity defined by Chinese criteria.

Table S1. Prevalence of Overweight by Region and Occupation (Chinese Criteria).

Occupational Category	Overall N=1427978	North n=1057723	South n=370255	P-value
Transportation, Storage, and Postal Services	35.32 (47.8)	36.14 (48.04)	30.66 (46.11)	<0.001
Accommodation and Catering Services	35.60 (47.89)	36.01 (48.01)	32.25 (46.78)	0.0596
Information Transmission, Software, and Information Technology Services	31.32 (46.38)	31.78 (46.56)	30.37 (45.98)	<0.001
Agriculture, Forestry, Animal Husbandry, and Fishery	37.59 (48.44)	38.14 (48.58)	28.14 (45.11)	0.0095
Manufacturing	34.13 (47.42)	35.26 (47.78)	32.32 (46.77)	<0.001
Health and Social Work	33.00 (47.02)	33.93 (47.35)	31.5 (46.45)	<0.001
Residential Services, Repair, and Other Services	34.27 (47.46)	33.74 (47.28)	36.25 (48.08)	0.0109
Construction	37.49 (48.41)	38.46 (48.65)	33.26 (47.12)	<0.001
Real Estate Industry	36.34 (48.1)	37.43 (48.39)	33.56 (47.22)	<0.001
Wholesale and Retail Trade	32.41 (46.8)	35.39 (47.82)	27.42 (44.61)	<0.001
Education	33.91 (47.34)	34.32 (47.48)	28.27 (45.1)	0.0238
Culture, Sports, and Entertainment	30.73 (46.14)	31.35 (46.39)	24.89 (43.25)	<0.001
Water Conservancy, Environment, and Public Facilities Management	37.81 (48.5)	38.02 (48.55)	33.75 (47.38)	0.1839
Production and Supply of Electricity, Heat, Gas, and Water	36.89 (48.25)	37.28 (48.36)	28.38 (45.16)	0.0019
Scientific Research and Technical Services	32.23 (46.73)	32.92 (46.99)	29.25 (45.49)	<0.001
Leasing and Business Services	32.95 (47)	33.66 (47.26)	29.88 (45.78)	<0.001
Mining Industry	34.62 (47.59)	34.57 (47.58)	42.86 (53.45)	0.6461
Financial Services	33 (47.02)	33.76 (47.29)	30.38 (45.99)	<0.001

Notes: Number of cases and corresponding prevalence rate (%).

Table S2. Prevalence of Obesity by Region and Occupation (Chinese Criteria).

Occupational Category	Overall N=1427978	North n=1057723	South n=370255	P-value
Transportation, Storage, and Postal Services	24.88 (43.23)	26.76 (44.27)	14.24 (34.95)	<0.001
Accommodation and Catering Services	18.68 (38.98)	18.94 (39.19)	16.51 (37.16)	0.1344
Information Transmission, Software, and Information Technology Services	13.76 (34.45)	15.18 (35.88)	10.83 (31.08)	<0.001
Agriculture, Forestry, Animal Husbandry, and Fishery	21.05 (40.77)	21.05 (40.78)	20.96 (40.82)	0.9768
Manufacturing	15.59 (36.28)	18.1 (38.5)	11.59 (32.01)	<0.001
Health and Social Work	15.64 (36.32)	17.9 (38.34)	11.97 (32.47)	<0.001
Residential Services, Repair, and Other Services	16.10 (36.76)	16.84 (37.43)	13.33 (33.99)	<0.001
Construction	21.04 (40.76)	22.07 (41.47)	16.5 (37.12)	<0.001
Real Estate Industry	20.95 (40.69)	22.87 (42)	16.02 (36.68)	<0.001
Wholesale and Retail Trade	16.49 (37.11)	19.84 (39.88)	10.88 (31.14)	<0.001
Education	15.66 (36.34)	16.04 (36.7)	10.42 (30.59)	0.0062
Culture, Sports, and Entertainment	14.08 (34.79)	14.52 (35.24)	9.86 (29.82)	<0.001
Water Conservancy, Environment, and Public Facilities Management	21.47 (41.07)	21.81 (41.3)	15 (35.78)	0.0123
Production and Supply of Electricity, Heat, Gas, and Water	26.63 (44.21)	27.24 (44.52)	13.51 (34.24)	<0.001
Scientific Research and Technical Services	14.37 (35.08)	15.06 (35.77)	11.41 (31.79)	<0.001
Leasing and Business Services	18.81 (39.08)	20.56 (40.42)	11.34 (31.71)	<0.001
Mining Industry	14.37 (35.09)	14.44 (35.16)	NA	NA
Financial Services	14.04 (34.74)	15.19 (35.89)	10.11 (30.14)	<0.001

Notes: Number of cases and corresponding prevalence rate (%).NA indicated missing data due to insufficient sample size.

Table S3. Occupational Patterns in Overweight and Obesity (Chinese Criteria).

Occupational Category	Overweight		Obesity	
	OR (95% CI)	AOR (95% CI)	OR (95% CI)	AOR (95% CI)
**Occupations**				
Transportation, Storage, and Postal Services	1.2*** (1.17,1.22)	0.97*** (0.95,0.99)	2.07*** (2.03,2.12)	1.66*** (1.62,1.70)
Accommodation and Catering Services	1.21*** (1.15,1.28)	0.93** (0.88,0.99)	1.44*** (1.35,1.54)	1.20*** (1.12,1.29)
Agriculture, Forestry, Animal Husbandry, and Fishery	1.32*** (1.23,1.42)	0.92** (0.85,0.99)	1.67*** (1.53,1.82)	1.19*** (1.09,1.31)
Manufacturing	1.14*** (1.12,1.15)	1.00 (0.98,1.01)	1.16*** (1.14,1.18)	1.09*** (1.07,1.11)
Health and Social Work	1.08*** (1.06,1.1)	0.98** (0.96,1)	1.16*** (1.13,1.19)	1.21*** (1.18,1.24)
Residential Services, Repair, and Other Services	1.14*** (1.1,1.18)	1.01 (0.97,1.05)	1.20*** (1.15,1.26)	1.11*** (1.06,1.17)
Construction	1.32*** (1.29,1.34)	1.00 (0.98,1.02)	1.67*** (1.63,1.71)	1.33*** (1.29,1.37)
Real Estate Industry	1.25*** (1.23,1.27)	1.03*** (1.01,1.05)	1.66*** (1.63,1.7)	1.45*** (1.42,1.48)
Wholesale and Retail Trade	1.05*** (1.03,1.07)	0.97*** (0.95,0.98)	1.24*** (1.21,1.27)	1.27*** (1.24,1.3)
Education	1.13*** (1.06,1.19)	0.99 (0.93,1.05)	1.16*** (1.08,1.26)	1.25*** (1.15,1.35)
Culture, Sports, and Entertainment	0.97 (0.95,1)	0.87*** (0.85,0.90)	1.03 (0.99,1.06)	1.04** (1,1.08)
Water Conservancy, Environment, and Public Facilities Management	1.33*** (1.26,1.41)	0.91*** (0.85,0.97)	1.71*** (1.6,1.84)	1.20*** (1.11,1.29)
Production and Supply of Electricity, Heat, Gas, and Water	1.28*** (1.22,1.35)	0.90*** (0.85,0.95)	2.27*** (2.15,2.4)	1.74*** (1.64,1.84)
Scientific Research and Technical Services	1.04*** (1.03,1.05)	0.99** (0.98,1.00)	1.05*** (1.04,1.07)	1.03*** (1.01,1.04)
Leasing and Business Services	1.08*** (1.06,1.09)	0.93*** (0.92,0.94)	1.45*** (1.43,1.47)	1.34*** (1.32,1.36)
Mining Industry	1.16*** (1.04,1.3)	0.93 (0.83,1.05)	1.05 (0.9,1.22)	0.93 (0.8,1.09)
Financial Services	1.08*** (1.07,1.09)	0.97*** (0.95,0.98)	1.02*** (1.01,1.04)	1.09*** (1.07,1.11)

Table S3. Occupational Patterns in Overweight and Obesity (Chinese Criteria) (Continued).

Occupational Category	Overweight		Obesity	
	OR (95% CI)	AOR (95% CI)	OR (95% CI)	AOR (95% CI)
**Male**	NO	2.40*** (2.38,2.41)	NO	2.45*** (2.43,2.48)
**Married**	NO	1.07*** (1.06,1.07)	NO	1.01 (1,1.02)
**Age group**	NO		NO	
30-39		1.45*** (1.44,1.47)		1.22*** (1.2,1.23)
40-49		2.04*** (2.02,2.07)		1.28*** (1.27,1.3)
50-59		2.59*** (2.56,2.63)		0.99 (0.98,1.01)
60-69		2.78*** (2.73,2.83)		0.77*** (0.75,0.79)
>=70		2.60*** (2.53,2.68)		0.59*** (0.57,0.61)
**South**	NO	0.93*** (0.92,0.94)	NO	0.63*** (0.63,0.64)
**Associated complications**	NO		NO	
Hypertension		1.00 (0.99,1.01)		3.07*** (3.03,3.11)
Prediabetes		1.08*** (1.07,1.1)		2.30*** (2.26,2.34)
Diabetes		1.01* (1.00,1.02)		1.72*** (1.7,1.74)
Constant	0.46*** (0.45,0.46)"	0.19*** (0.19,0.19)	0.16*** (0.16,0.16)	0.07*** (0.07,0.07)
R^2	0.0009	0.0507	0.0069	0.0896
Observations	1427978	1427978	1427978	1427978

Notes: Regressions of column 2 and 4 controlled for gender, marital status, age, region and associated complications. *** p<0.01, ** p<0.05, * p<0.1.

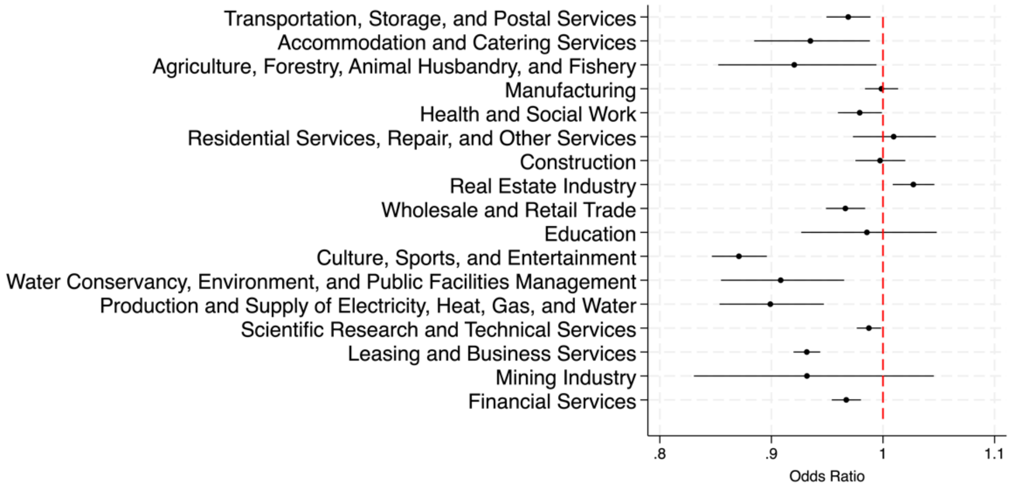

Figure S1. Adjusted Odds Ratio of Overweight across Occupations (Chinese Criteria).

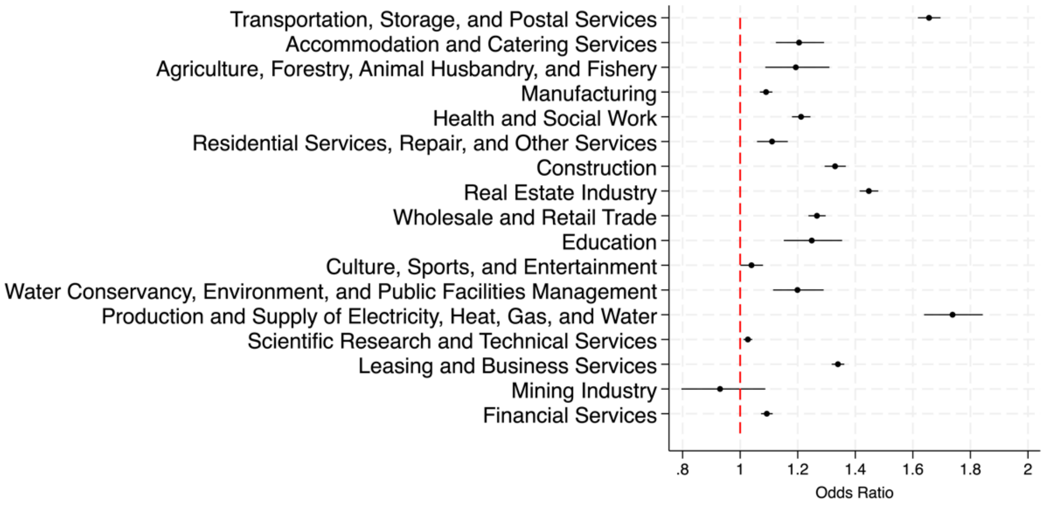

Figure S2. Adjusted Odds Ratio of Obesity across Occupations (Chinese Criteria).

Table S4. Association between Occupation and Obesity or Overweight (Chinese Criteria).

Occupational Category	Overweight		Obesity	
	OR (95% CI)	OR (95% CI)	OR (95% CI)	OR (95% CI)
Blue-Collar	1.16*** (1.14,1.17)	1.00 (0.99,1.01)	1.45*** (1.43,1.47)	1.24*** (1.22,1.26)
Sales/Office	1.07*** (1.06,1.08)	0.97*** (0.96,0.98)	1.40*** (1.39,1.42)	1.3*** (1.28,1.31)
Service	1.02* (1,1.04)	0.93*** (0.91,0.95)	1.10*** (1.07,1.13)	1.04*** (1.01,1.07)
Other covariates	NO	YES	NO	YES
R^2	0.0005	0.0506	0.0045	0.0885
Observations	1427978	1427978	1427978	1427978

Notes: Management/Professional as the control group. *** p<0.01, ** p<0.05, * p<0.1.

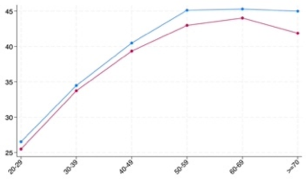

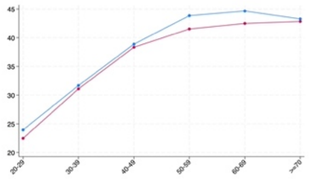

(1) (2)

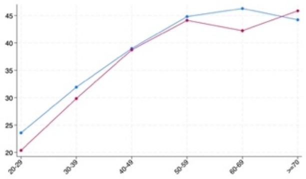

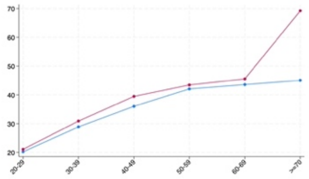

(3) (4)

Figure S3. Prevalence of Overweight across Region and Age (Chinese Criteria).

Notes: The blue line denotes north and the red line denotes south. **(1)** Overweight of Blue-collar. **(2)** Overweight of Management/Professional. **(3)** Overweight of Sales/Office. **(4)** Overweight of Service.

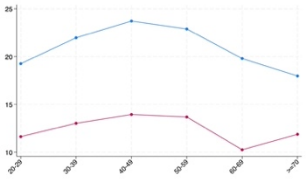

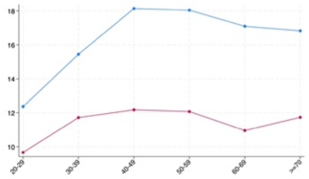

(1) (2)

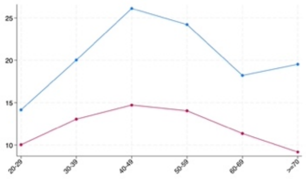

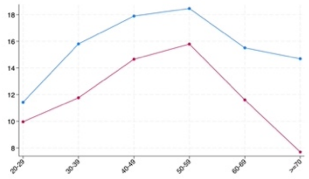

(3) (4)

Figure S4. Prevalence of Obesity across Region and Age (Chinese Criteria).

Notes: The blue line denotes north and the red line denotes south. **(1)** Obesity of Blue-collar. **(2)** Obesity of Management/Professional. **(3)** Obesity of Sales/Office. **(4)** Obesity of Service.
